# Prefrontal Lobe and Posterior Cingulate Cortex Activations in Patients with Major Depressive Disorder by Using Standardized Weighted Low-Resolution Electromagnetic Tomography

**DOI:** 10.3390/jpm11111054

**Published:** 2021-10-21

**Authors:** I-Mei Lin, Hong-En Yu, Yi-Chun Yeh, Mei-Feng Huang, Kuan-Ta Wu, Chiao-Li Khale Ke, Pei-Yun Lin, Cheng-Fang Yen

**Affiliations:** 1 Department of Psychology, College of Humanities and Social Sciences, Kaohsiung Medical University, Kaohsiung City 80708, Taiwan; 2 Department of Medical Research, Kaohsiung Medical University Hospital, Kaohsiung City 80708, Taiwan; 3 Department of Psychiatry, Kaohsiung Medical University Hospital, Kaohsiung Medical University, Kaohsiung City 80708, Taiwan; y7552156@gmail.com (Y.-C.Y.); lalalabon@gmail.com (M.-F.H.); koakhale@gmail.com (C.-L.K.K.); parin520@gmail.com (P.-Y.L.); p03132006@gmail.com (C.-F.Y.); 4 Graduate Institute of Medicine, College of Medicine, Kaohsiung Medical University, Kaohsiung City 80708, Taiwan; 5 Health Management Center, Kaohsiung Medical University Hospital, Kaohsiung Medical University, Kaohsiung City 80708, Taiwan; 990008@mail.kmuh.org.tw; 6 Department of Psychiatry, Kaohsiung Municipal SiaoGang Hospital, Kaohsiung Medical University, Kaohsiung City 80708, Taiwan

**Keywords:** major depressive disorder, standardized weighted low-resolution electromagnetic tomography (swLORETA), electroencephalography

## Abstract

Background: The differences in brain activity between patients with major depressive disorder (MDD) and healthy adults have been confirmed by functional magnetic resonance imaging (fMRI), positron emission tomography (PET), and electroencephalography (EEG). The prefrontal lobe and posterior cingulate cortex (PCC) are related to emotional regulation in patients with MDD. However, the high cost and poor time resolution of fMRI and PET limit their clinical application. Recently, researchers have used high time resolution of standardized weighted low-resolution electromagnetic tomography (swLORETA) to investigate deep brain activity. This study aimed to convert raw EEG signals into swLORETA images and explore deep brain activity in patients with MDD and healthy adults. Methods: BrainMaster EEG equipment with a 19-channel EEG cap was used to collect resting EEG data with eyes closed for 5 min. NeuroGuide software was used to remove the EEG artifacts, and the swLORETA software was used to analyze 12,700 voxels of current source density (CSD) for 139 patients with MDD and co-morbid anxiety symptoms (mean age = 43.08, SD = 13.76; 28.78% were male) and 134 healthy adults (mean age = 40.60, SD = 13.52; 34.33% were male). Deep brain activity in the frontal lobe and PCC at different frequency bands was analyzed, including delta (1–4 Hz), theta (5–7 Hz), alpha (8–11 Hz), beta (12–24 Hz), beta1 (12–14 Hz), beta2 (15–17 Hz), beta3 (18–24 Hz), and high beta (25–29 Hz). Results: There was lower delta and theta and higher beta, beta1, beta2, beta3, and high-beta activity at the prefrontal lobe (dorsal medial prefrontal cortex [dmPFC], ventral medial prefrontal cortex [vmPFC], and dorsal lateral prefrontal cortex [dlPFC], ventral lateral prefrontal cortex [vlPFC], orbital frontal cortex [OFC]) and PCC in MDD patients compared with healthy adults. There was no significant difference in alpha activity between the two groups. Conclusion: This study indicates brain hyperactivity in the right prefrontal lobe (dlPFC and vmPFC) and PCC in patients with MDD with co-morbid anxiety symptoms, and the dlPFC and PCC were also related to emotion regulation in MDD. Inhibiting high-beta activity or restoring delta and theta activity to the normal range in the right frontal lobe and PCC may be possible in z-score neurofeedback protocols for patients with MDD in future studies.

## 1. Introduction

According to the World Health Organization, 264 million people worldwide suffer from depressive disorder, which has become the second most disabling and burdensome disease in the world [1]. Gutiérrez-Rojas et al. [2] systematically reviewed 63 studies reporting the prevalence rates for depression and found that the lifetime prevalence rate was between 2 and 21%, with an average of 11.32%, and the 12-month prevalence rate was 5.2%.

The pathophysiology of electroencephalography (EEG) in depression includes frontal alpha asymmetry [3], parietal lobe hyperactivity in patients with co-morbid depression and anxiety [4,5], and lower delta and theta waves with higher beta waves in the whole brain area [6]. Studies of functional magnetic resonance imaging (fMRI) and positron emission tomography (PET) found that patients with depression have lower activation in the left frontal lobe or left amygdala [7] and higher activation in the right dorsal lateral prefrontal cortex (dlPFC; BA8, BA9, BA10, and BA46) compared with healthy controls [8]. However, the high cost and side effects of radiation for fMRI and PET limit their clinical use. In recent years, the development and updating of brain imaging technology for transforming surface EEG to current source density (CSD) to investigate deep brain activity was improved. The development of low-resolution electromagnetic tomography (LORETA) [9] and standardized LORETA (sLORETA) [10] progressed to standardized weighted LORETA (swLORETA), which divides the brain into 12,700 voxels so that locating the current source signal can be improved and the accuracy can be increased. The correctness of LORETA brain imaging has been verified with fMRI [11] and PET scans [12,13]. Because of the convenience and effectiveness of LORETA, which can avoid the side effects caused by fMRI and PET, it can be beneficial in diagnosis and research for patients with depression.

The frontal lobe, parietal lobes, and posterior cingulate cortex (PCC) are related to emotional regulation in depression. The frontal lobe includes the medial prefrontal cortex (mPFC; BA8, BA9, BA10, BA11, BA12, BA25), dorsolateral prefrontal cortex (dlPFC; BA8, BA9, BA10, BA46), ventrolateral prefrontal cortex (vlPFC, BA44, BA45, and BA47), and orbitofrontal prefrontal cortex (OFC; BA10 and BA11). The mPFC is divided into the dorsomedial prefrontal cortex (dmPFC; BA8 and BA9, related to emotional regulation, self-referential processes, rumination, and cognitive reappraisal), and ventral medial prefrontal cortex (vmPFC; BA10, BA11, BA13, BA32, related to positive emotional regulation and social cognition) [14].

EEG, fMRI, and EEG were applied to explore brain activity in the left and right PFC and the PCC among patients with MDD. (1) Hypoactivity in left PFC: Lubar et al. [15] found higher alpha2 asymmetry in the middle frontal gyrus (BA6) and medial frontal gyrus (BA10) in the MDD group. Saletu et al. [16], in a LORETA study, found that depression in women showed lower theta and alpha1 waves in the vmPFC compared to the HC group, and theta waves were negatively correlated with the depression scale. Auerbach et al. [17] used LORETA and found that young girls with depression had higher theta and alpha CSD in the left dlPFC than healthy young girls. The same result of lower brain metabolic rate and lower blood flow in the left prefrontal lobe than the right prefrontal lobe was supported by PET and fMRI studies [18,19,20]. These results indicate hypoactivity in the left prefrontal lobe compared to the right prefrontal lobe, and that the left dlPFC plays an important role in the regulation of positive and negative emotions. (2) Hyperactivity in right PFC: Patients with depression had higher beta3 activity (21.5–30.0 Hz) in the right dlPFC (superior frontal gyrus; BA9, BA10) and higher activation in the right OFC (inferior frontal gyrus; BA11) as determined by measuring the Fz, F4, and AFz of surface EEG transformed to CSD [8], as well as higher CSD at the right dlPFC (AF4 and F6) compared with healthy controls [21]. Saletu et al. [16] also reported hyperactivity in the right vmPFC in depression. PET and fMRI studies also showed higher metabolic rates and blood flow in the right prefrontal lobe, such as right dlPFC (BA9/46), in patients with depression compared with healthy controls [8,18,19]. These results indicate hyperactivity in the right prefrontal in patients with depression compared with healthy controls. (3) Abnormal activation in the PCC: Koberda [22] found abnormal activation in the PCC, subcallosal cingulate (BA25), and OFC (BA11) in 31 patients with depression with co-morbid anxiety and without anxiety. Pizzagalli et al. [21] also found hypoactivation in the PCC in patients with depression, including lower alpha1 activity (8.5–10.0 Hz) in the bilateral PCC (BA31), lower beta3 activity in the bilateral PCC (BA23 and BA31) and the posterior-anterior cuneiform lobe (BA7). However, Takamura et al. [23] found that patients with co-morbid MDD and anxiety had more highly overactivated PCC, which may be related to their rumination symptoms. Therefore, hypoactivity in the left prefrontal lobe, hyperactivity in the right prefrontal lobe, and abnormal activity in the PCC may be related to the pathophysiology of depression.

While previous studies have converted EEG to LORET to reflect deep brain activity, research with rigorous design and large samples is still lacking. The purpose of this study was to use swLORETA to investigate deep brain activity in patients with MDD and healthy controls, especially focusing on the prefrontal lobe and posterior cingulate gyrus related to emotion regulation.

## 2. Methods

### 2.1. Participants

Patients with MDD with co-morbid anxiety disorder or anxiety symptoms (MDD group) were referred by psychiatrists from Kaohsiung Medical University Hospital, Kaohsiung Municipal Ta-Tung Hospital, Kaohsiung Chang Gung Memorial Hospital, and Kaohsiung Municipal Siaogang Hospital. The inclusion criteria of MDD were as follows: (1) primary diagnosis of MDD based on DSM-5 (patients with co-morbid MDD and anxiety disorder were also enrolled as long as the primary diagnosis was MDD); (2) Beck Depression Inventory-II (BDI–II) and Beck Anxiety Inventory (BAI) scores higher than 14 and 8; and (3) age 20–75 years. Exclusion criteria were as follows: (1) diagnosis of mental disorder other than MDD (e.g., bipolar disorder, substance use, schizophrenia, etc.), and (2) MDD due to other medical conditions (e.g., cancer, kidney disease, stroke, etc.).

The HC group was recruited from the Health Management Center of Kaohsiung Medical University Hospital, the campus of Kaohsiung Medical University, and the community of Kaohsiung City. The inclusion criteria were as follows: (1) no severe physical illness (e.g., cancer, kidney disease, stroke, etc.) or mental disorder (e.g., depressive disorder, anxiety disorder, bipolar disorder, substance use, schizophrenia, etc.), and (2) BDI–II and BAI scores lower than 14 and 8.

A total of 179 patients with MDD and 188 healthy controls were enrolled for EEG assessment. In the MDD group, one patient with a co-morbid alcohol-related disorder was ruled out from data analysis, four EEG files were damaged, and the files of 35 patients with eye blinking and movement artifacts in one of the channels were deleted, because swLORETA needs 19 channels to convert to deep brain activity. In the HC group, 51 participants had eye blinking and movement in one of the channels, and three of them took medicine for co-morbid diabetes and refused to finish the EEG measurement and were removed from data analysis. Finally, 139 patients with MDD and 134 healthy controls were analyzed by swLORETA.

The study protocol was approved by the Ethics Committee of Kaohsiung Medical University Hospital (KMUH-IRB-2012-02-09-II, KMUH-IRB-F-I-20160027, and KMUHIRB-F-I-20200117) and Kaohsiung Chang Gung Memorial Hospital (CGMH IRB: 1604250002). Each participant provided informed consent and received NT 300 (about USD 11) for their participation.

### 2.2. Psychological Questionnaires

Demographic data (e.g., age, sex), BDI-II depression score, and BAI anxiety score were collected. The BDI-II includes 21 items scored on a 4-point Likert scale to assess depressive symptoms, and the total score ranges from 0 to 63 [24]. A score between 0 and 13 is considered as normal, 14 to 19 as mild depression, 20 to 28 as moderate depression, and 29 to 63 as severe depression. Cronbach’s α for outpatients is 0.92 and one-week test-retest reliability is 0.93. The correlation between the BDI-II and the Beck Hopelessness Scale is 0.68, and the correlation between the BDI-II and the Scale for Suicide Ideation is 0.37 [24]. The Chinese version of BDI-II was translated by Chen [25], for which Cronbach’s α is 0.94, split-half reliability is 0.91, and the correlation between the BDI-II and the Chinese Health Questionnaire is 0.69 [26]. The BAI includes 21 items scored on a 4-point Likert scale to assess anxiety symptoms, and the total score ranges from 0 to 63 [27]. A total score of 0 to 7 is considered as normal, 8 to 15 as mild anxiety, 16 to 25 as moderate anxiety, and 26 to 63 as severe anxiety. The Cronbach’s α for outpatients is 0.92 and one-week test-retest reliability is 0.75. The correlation between the BDI-II and the Hamilton Anxiety Rating Scale is 0.51 [24]. The Chinese version of BDI-II was translated by Lin [28], for which Cronbach’s α is 0.95 and split-half reliability is 0.91, and the correlation between BAI and the Hamilton Anxiety Rating Scale is 0.72 [29].

### 2.3. EEG Equipment and Measurement

BrainAvatar version 4.0 with an impedance lid amplifier (BrainMaster Technologies, Inc., Bedford, OH, USA) connected to a 19-channel EEG cap was used to collect 5 min resting EEG based on the international 10–20 system, including Fp1, Fp2, Fz, F3, F4, F7, F8, Cz, C3, C4, T3, T4, T7, T8, Pz, P3, P4, O1, and O2. A linked-ear reference was used, and the impedance was maintained below 5 kΩ. A band-pass filter between 0 to 100 Hz was applied, and the notch was filtered at 60 Hz, with a sampling rate of 256 Hz.

### 2.4. EEG Data Processing

The EEG data was exported into NeuroGuide software (Applied Neuroscience, Inc, St. Petersburg, FL, USA), and the researchers examined movement and eye blink artifacts in a 20 s window. The clean EEG data were analyzed by NeuroNavigator software (Applied Neuroscience, Inc, St. Petersburg, FL, USA) and transformed to swLORETA for CSD. If EEG artifacts in one channel were higher than 150 s, the data were rejected for statistical analysis. The EEG data were transformed to CSD at dmPFC, vmPFC, dlPFC, vlPFC, OFC, and PCC for the following frequency bands: delta (1–4 Hz), theta (4–8 Hz), alpha (8–12 Hz), beta1 (12–14 Hz), beta2 (15–17 Hz), beta3 (18–24 Hz), and high-beta (25–29 Hz).

### 2.5. Statistical Analysis

Statistical analyses were performed using SPSS software version 21.0 (IBM Corporation, Armonk, NY, USA). The *t*-test and chi-squared test were used to examine the differences in demographic data, depression, and anxiety between the MDD and HC groups. Multivariate analysis of variance (MANOVA) was used to examine brain activity at the dmPFC, vmPFC, dlPFC, vlPFC, OFC, and PCC between the MDD and HC groups.

## 3. Results

### 3.1. Participant Characteristic and Psychological Questionnaires of MDD and HC Groups

A total of 139 patients with MDD (mean age = 43.079 years, SD = 13.758; 28.78% male) and 134 healthy controls (mean age = 40.604 years, SD = 13.515; 34.33% male) were included in the statistical analysis. There were no significant differences in age and sex between the MDD and HC groups (*t*_(271)_ = 1.499, *p* = 0.135; and *χ_(1)_*^2^ = 0.974, *p* = 0.324). There were higher total BDI-II (*t*_(168.412)_ = 30.348, *p* < 0.001) and BAI (*t*_(148.818)_ = 24.562, *p* < 0.001) scores in the MDD group compared to the HC group (Table 1).

### 3.2. Deep Brain Activity in PFC and PCC between MDD and HC Groups

**Figure 1:**
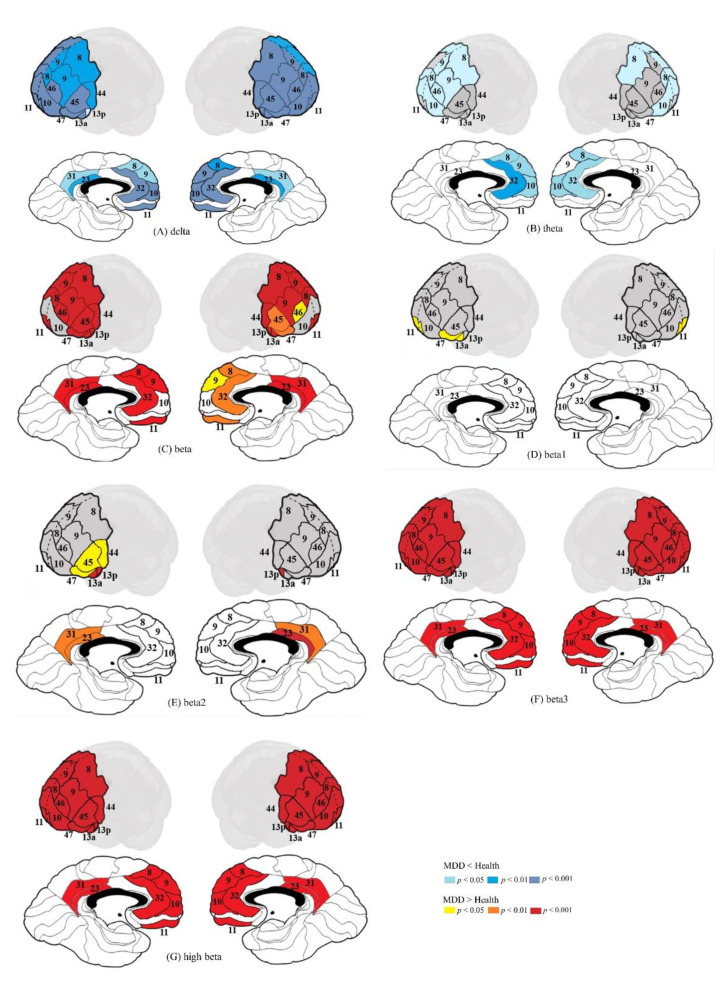
Deep brain activity of different frequency bands for MDD and HC groups: (**A**) delta; (**B**) theta; (**C**) beta; (**D**) beta1; (**E**) beta2; (**F**) beta3; (**G**) high beta.

The results of multivariate analysis showed that the MDD group had lower delta and theta and higher beta (especially beta3 and high beta) activity than the HC group in dmPFC, vmPFC, dlPFC, vlPFC, OFC, and PCC. The detailed information is as follows (Table 2 and Figure 1): (1) dmPFC: The MDD group had lower delta and theta and higher beta (especially beta3 and high-beta) activity than the HC group in dmPFC, including L-BA8, R-BA8, L-BA9, and R-BA9; (2) vmPFC: The MDD group had lower delta and theta and higher beta activity than the HC group in vmPFC, including lower delta and theta in L-BA10, R-BA10, L-BA 32, and R-BA32; lower delta in L-BA11, R-BA11, L-BA13a, R-BA13a, L-BA13p, and R-BA13p; higher beta3 and high-beta in L-BA10, R-BA10, L-BA11, R-BA11, L-BA13a, R-BA13a, L-BA13p, R-BA13p, L-BA32, and R-BA32; and higher beta1 in L-BA11; higher beta1 and beta2 in L-BA13a; and higher beta2 in L-BA13p and R-BA13p; (3) dlPFC: The MDD group had lower delta and theta and higher beta activity than the HC group in dlPFC, including lower delta in L-BA8, L-BA9, L-BA10, L-BA46, R-BA8, R-BA9, R-BA10, and R-BA46; lower theta in L-BA8, L-BA9, L-BA10, L-BA46, R-BA8, R-BA9, and R-BA10; and higher beta3 and high-beta in L-BA8, L-BA9, L-BA10, L-BA46, R-BA8, R-BA9, R-BA10, and R-BA46; (4) vlPFC: The MDD group had lower delta and higher beta (especially beta3 and high-beta) activity than the HC group in vlPFC, including in L-BA44, L-BA45, L-BA47, R-BA44, R-BA45, and R-BA47; higher beta1 in L-BA47; and higher beta2 in L-BA44, L-BA45, and L-BA47; (5) OFC: The MDD group had lower delta activity than the HC group in L-BA10, L-BA11, R-BA10, and R-BA11; lower theta in L-BA10 and R-BA10; higher beta (beta3 and high-beta) in L-BA10, L-BA11, R-BA10, and R-BA11; and higher beta1 in L-BA11; and (6) PCC: The MDD group had lower delta and higher beta (beta2, beta3, and high-beta) activity than the HC group in L-BA23, L-BA31, R-BA23, and R-BA31.

## 4. Discussion

This study used swLORETA to analyze surface EEG representing deep brain activity. The results show that the MDD group had hyperactivity in the PFC (including dmPFC, vmPFC, dlPFC, vlPFC, and OFC) and PCC at resting baseline compared to the HC group; the hyperactivation was reflected in lower delta and theta and higher beta3 and high-beta activity. Our results supported the hypothesis that functional and structural changes in emotional regulation among patients with MDD, especially in vmPFC, OFC, dlPFC, ACC, ventral striatum, amygdala, and hippocampus, results in disrupted reciprocal connections between the prefrontal cortex and limbic area. This results in an overactive limbic area and dysfunction of the prefrontal cortex’s ability to regulate emotion and stress response [30]. The cortical–striatal–pallidal–thalamic circuit contains PFC, ACC, basal ganglia, and thalamus that are related to sensory information, cognitive processes, and emotional regulation among patients with MDD [31,32]. Decreased gray matter volume was observed in the insula, putamen, amygdala, lingual gyrus, and cerebellum; these brain areas were located within the cortico-striatal-pallidal-thalamic circuit [33]. A meta-analysis study indicated a link between the significantly decreased volume in the prefrontal cortex (especially orbitofrontal) and ACC, as well as in subcortical structures such as the caudate nucleus and putamen [31]. Connolly et al. also reported that patients with MDD had lower connectivity between the PFC (dlPFC, vmPFC, vlPFC) and the amygdala compared to healthy controls, and that lower connectivity indicated hyperactivity in PFC and failed to regulate amygdala activity in emotional regulation [34].

### 4.1. Hyperactivation in PFC

The results of this study are consistent with previous findings in LORETA studies. Pizzagalli et al. [21] used LORETA to analyze deep brain activity and found higher beta3 activity in the right dlPFC in people with depression compared to healthy controls; there were significant positive correlations between beta3 of the right dlPFC and depression and anxiety. Korb et al. [35] reported similar results of a LORETA study showing that patients with depression had higher beta activity in the dlPFC compared to the HC group and prefrontal cortex hyperactivity. A magnetoencephalogram study found that patients with MDD decreased prefrontal–amygdala connectivity, and results in dlPFC failed to regulate amygdala activity in countering sadness stimuli [36]. An fMRI study also indicated the positive influence from bilateral ACC to dmPFC, and from insula to dmPFC under the emotion stimuli in healthy controls; however, MDD patients had higher pulvinar activity and weakened reciprocal pathways between the insula and dmPFC, and this caused the dmPFC hyperactivity. While dmPFC failed to regulate pulvinar activity, it may influence visual information processing between the visual cortex and pulvinar, as well as between the pulvinar and insula [37]. In another LORETA study, Saletu et al. [16] also reported hypoactivity in the left vmPFC and hyperactivity in the right vmPFC among female patients with depression compared to the HC group, and theta activity was negatively correlated with the depression scale; that is, the vmPFC was more highly activated in patients with depression. This result was consistent with an fMRI study showing higher activity in the PFC. Herrington et al. [8] found that patients with co-morbid depression and anxiety symptoms had higher activity in the right dlPFC than healthy controls. This study not only found excessive activity in the dlPFC and vmPFC, but also similar results in the dmPFC, vlPFC, and OFC; that is, MDD patients were shown to have excessive activation of the entire prefrontal lobe.

### 4.2. Hyperactivation in PCC

This study found that the left and right PCCs showed a high activation state, which was consistent with the results reported by Takamura et al. [23] and inconsistent with the low activation of PCC (low beta3) reported in [21]. A retrospective study by Koo et al. [38] found that the right prefrontal lobe and right posterior parietal lobe may be related to more intense unpleasant emotions in patients with depression. Penner et al. [39] indicated that the left pulvinar connected to precuneus, PCC, middle cingulate, left dlPFC, dmPFC, and left inferior occipital regions, and the right pulvinar connected to the precuneus, middle cingulate, dmPFC, and left superior frontal regions in the resting state of healthy controls. However, the patients with MDD showed low connectivity between the left pulvinar and precuneus, middle cingulate, and left dlPFC. The PCC hyperactivity may be related to rumination symptoms in patients with co-morbid MDD and anxiety [23]. These inconsistent results may be due to the depression subtypes or heterogeneity and do not distinguish between depression and co-morbid anxiety, suicide, rumination, and other symptoms [23,40,41]. Moreover, some studies only included one-month of unmedicated depression in female patients [15] and one-week medication-free depression [18].

Studies have found that the PCC is an important hub of the default mode network (DMN), which is related to self-reference and rumination in the resting state. In a meta-analysis, hyperactivation between dlPFC and DMN was found to be related to depression [14]. Some studies have applied neurofeedback to increase activation of dlPFC and reduce activation of PCC, and the results showed that it can reduce depressive symptoms in patients with depression with co-morbid anxiety [22]. Poor functional regulation and emotional modulation of the PFC and PCC were found in patients with co-morbid depression and anxiety; the hyperactivity of the PFC and PCC may be related to the pathophysiology of depression [42].

### 4.3. Limitations

There were two limitations in this study. First, patients with MDD were taking prescription medications, and we did not ask them to stop their medication before EEG assessment. The results cannot rule out medication effects in higher beta (beta3 and high beta) activation. Second, this study did not distinguish the subtypes, and did not examine the deep brain activity of PFC and PCC regarding the heterogeneity of depression.

### 4.4. Summary

The present study demonstrated higher activation in PFC (including dlPFC, dmPFC, vlPFC, vmPFC, and OFC) and PCC in patients with co-morbid depression and anxiety symptoms. swLORETA was used to analyze surface EEG and convert it to deep brain activation, and the results are consistent with fMRI and PET studies. The results indicate that PFC and PCC overactivation may be the underlying pathophysiology of depression. swLORETA neurofeedback was developed in recent years and could be applied to decrease PFC and PCC activation in future studies.

## Figures and Tables

**Table 1:** Demographic data, BDI-II and BAI scores, and medications for MDD (*n* = 139) and HC (*n* = 134) groups.

	MDD Group Mean (SD)	HC Group Mean (SD)	*t/χ^2^*	*p*
Age	43.079 (13.758)	40.604 (13.515)	1.499	0.135
Sex Male	46 (34.33%)	40 (28.78%)	0.974	0.324
Female	88 (65.67%)	99 (71.22%)		
Total BDI-II score	4.000 (3.376)	31.907 (10.281)	30.348 ***	<0.001
Total BAI score	2.015 (1.943)	23.216 (9.982)	24.562 ***	<0.001
Duration of MDD history (years)	6.651 (7.040)	NA		
Duration of prescriptions (years)	6.755 (7.060)	NA		
Medications, *n* (%)				
Antidepressants	125 (89.93%)	NA		
Selective serotonin reuptake inhibitors (SSRIs)	49 (35.25%)	NA		
Serotonin and norepinephrine reuptake inhibitors (SNRIs)	19 (13.67%)	NA		
Tricyclic antidepressants	7 (5.04%)	NA		
Other antidepressants	69 (49.64%)	NA		
Benzodiazepine	98 (70.50%)	NA		
Hypnotics/sedatives	27 (19.42%)	NA		
Without medication or missing	14 (10.07%)	NA		

*** *p* < 0.001 BAI, Beck Anxiety Inventory; BDI-II, Beck Depression Inventory-II.

**Table 2:** Deep brain activity in dmPFC, vmPFC, dlPFC, vlPFC, OFC, and PCC for MDD and HC groups.

**dmPFC**		**MDD Group** **Mean (SD)**	**HC Group** **Mean (SD)**	***F***	***p***
L-BA8	delta	0.0000140 (0.00001081)	0.0000194 (0.00001890)	8.495 **	<0.01
	theta	0.0000047 (0.00000326)	0.0000056 (0.00000315)	5.838 *	<0.05
	alpha	0.0000054 (0.00000442)	0.0000058 (0.00000473)	0.540	0.463
	beta	0.0000039 (0.00000358)	0.0000025 (0.00000278)	12.572 ***	<0.001
	beta1	0.0000029 (0.00000213)	0.0000026 (0.00000173)	1.826	0.178
	beta2	0.0000037 (0.00000342)	0.0000032 (0.00000877)	0.274	0.601
	beta3	0.0000044 (0.00000478)	0.0000022 (0.00000251)	23.382 ***	<0.001
	high-beta	0.0000032 (0.00000329)	0.0000017 (0.00000181)	22.357 ***	<0.001
R-BA8	delta	0.0000126 (0.00000985)	0.0000177 (0.00001267)	13.711 ***	<0.001
	theta	0.0000046 (0.00000333)	0.0000055 (0.00000303)	5.284 *	<0.05
	alpha	0.0000055 (0.00000466)	0.0000057 (0.00000377)	0.149	0.700
	beta	0.0000040 (0.00000361)	0.0000026 (0.00000275)	11.803 **	0.001
	beta1	0.0000029 (0.00000217)	0.0000026 (0.00000172)	1.571	0.211
	beta2	0.0000039 (0.00000410)	0.0000036 (0.00000855)	0.183	0.669
	beta3	0.0000044 (0.00000474)	0.0000022 (0.00000254)	22.797 ***	<0.001
	high-beta	0.0000031 (0.00000321)	0.0000017 (0.00000185)	20.365 ***	<0.001
L-BA9	delta	0.0000168 (0.00001246)	0.0000234 (0.00002248)	9.166 **	<0.01
	theta	0.0000051 (0.00000352)	0.0000060 (0.00000329)	5.190 *	<0.05
	alpha	0.0000064 (0.00000527)	0.0000067 (0.00000531)	0.288	0.592
	beta	0.0000040 (0.00000360)	0.0000026 (0.00000268)	13.876 ***	<0.001
	beta1	0.0000030 (0.00000211)	0.0000027 (0.00000164)	2.053	0.153
	beta2	0.0000040 (0.00000378)	0.0000035 (0.00000855)	0.401	0.527
	beta3	0.0000045 (0.00000480)	0.0000022 (0.00000242)	24.858 ***	<0.001
	high-beta	0.0000033 (0.00000326)	0.0000017 (0.00000181)	25.316 ***	<0.001
R-BA9	delta	0.0000154 (0.00001138)	0.0000221 (0.00001326)	20.055 ***	<0.001
	theta	0.0000048 (0.00000370)	0.0000057 (0.00000301)	4.172 *	<0.05
	alpha	0.0000062 (0.00000539)	0.0000062 (0.00000411)	0.016	0.900
	beta	0.0000042 (0.00000378)	0.0000028 (0.00000367)	8.502 **	<0.01
	beta1	0.0000030 (0.00000204)	0.0000027 (0.00000169)	1.662	0.198
	beta2	0.0000046 (0.00000679)	0.0000044 (0.00001327)	0.039	0.844
	beta3	0.0000045 (0.00000477)	0.0000023 (0.00000264)	22.254 ***	<0.001
	high-beta	0.0000032 (0.00000326)	0.0000018 (0.00000195)	20.664 ***	<0.001
**vmPFC**		**MDD group** **Mean (SD)**	**Healthy controls** **Mean (SD)**	***F***	***p***
L-BA10	delta	0.0000210 (0.00001396)	0.0000314 (0.00002238)	21.191 ***	<0.001
	theta	0.0000057 (0.00000388)	0.0000067 (0.00000343)	5.020 *	<0.05
	alpha	0.0000070 (0.00000678)	0.0000066 (0.00000479)	0.241	0.624
	beta	0.0000040 (0.00000393)	0.0000029 (0.00000514)	4.544	0.157
	beta1	0.0000029 (0.00000197)	0.0000026 (0.00000176)	1.399	0.238
	beta2	0.0000053 (0.00001128)	0.0000050 (0.00002009)	0.025	0.875
	beta3	0.0000040 (0.00000396)	0.0000021 (0.00000243)	23.594 ***	<0.001
	high-beta	0.0000032 (0.00000365)	0.0000017 (0.00000201)	17.85 ***	<0.001
R-BA10	delta	0.0000208 (0.00001390)	0.0000315 (0.00002064)	25.500 ***	<0.001
	theta	0.0000056 (0.00000426)	0.0000066 (0.00000339)	4.147 *	<0.05
	alpha	0.0000066 (0.00000637)	0.0000061 (0.00000409)	0.574	0.446
	beta	0.0000043 (0.00000447)	0.0000031 (0.00000597)	3.824	0.052
	beta1	0.0000030 (0.00000205)	0.0000027 (0.00000193)	1.701	0.193
	beta2	0.0000059 (0.00001361)	0.0000056 (0.00002325)	0.017	0.895
	beta3	0.0000043 (0.00000434)	0.0000022 (0.00000288)	20.924 ***	<0.001
	high-beta	0.0000033 (0.00000360)	0.0000018 (0.00000222)	16.964 ***	<0.001
L-BA11	delta	0.0000220 (0.00001220)	0.0000297 (0.00001663)	19.136 ***	<0.001
	theta	0.0000074 (0.00000577)	0.0000080 (0.00000434)	0.969	0.326
	alpha	0.0000145 (0.00001754)	0.0000127 (0.00001219)	1.006	0.317
	beta	0.0000048 (0.00000385)	0.0000030 (0.00000346)	16.495 ***	<0.001
	beta1	0.0000043 (0.00000305)	0.0000036 (0.00000215)	5.429 *	<0.05
	beta2	0.0000060 (0.00000806)	0.0000043 (0.00001287)	1.622	0.204
	beta3	0.0000046 (0.00000409)	0.0000022 (0.00000190)	35.659 ***	<0.001
	high-beta	0.0000032 (0.00000333)	0.0000016 (0.00000148)	25.599 ***	<0.001
R-BA11	delta	0.0000196 (0.00001077)	0.0000277 (0.00001535)	25.469 ***	<0.001
	theta	0.0000069 (0.00000550)	0.0000075 (0.00000408)	1.195	0.275
	alpha	0.0000128 (0.00001469)	0.0000112 (0.00001047)	1.000	0.318
	beta	0.0000047 (0.00000398)	0.0000030 (0.00000373)	12.939 ***	<0.001
	beta1	0.0000040 (0.00000288)	0.0000033 (0.00000207)	4.656	0.031
	beta2	0.0000059 (0.00000913)	0.0000045 (0.00001368)	1.053	0.306
	beta3	0.0000044 (0.00000419)	0.0000022 (0.00000225)	29.511 ***	<0.001
	high-beta	0.0000031 (0.00000334)	0.0000016 (0.00000165)	20.062 ***	<0.001
L-BA13a	delta	0.0000226 (0.00001448)	0.0000287 (0.00001524)	11.453 ***	<0.001
	theta	0.0000080 (0.00000616)	0.0000088 (0.00000480)	1.383	0.241
	alpha	0.0000172 (0.00001864)	0.0000160 (0.00001330)	0.363	0.547
	beta	0.0000055 (0.00000424)	0.0000033 (0.00000215)	27.450 ***	<0.001
	beta1	0.0000052 (0.00000382)	0.0000043 (0.00000260)	4.861 *	<0.05
	beta2	0.0000061 (0.00000552)	0.0000040 (0.00000470)	10.846 ***	0.001
	beta3	0.0000054 (0.00000475)	0.0000027 (0.00000202)	37.623 ***	<0.001
	high-beta	0.0000037 (0.00000340)	0.0000019 (0.00000144)	33.132 ***	<0.001
R-BA13a	delta	0.0000164 (0.00000994)	0.0000231 (0.00001266)	23.835 ***	<0.001
	theta	0.0000061 (0.00000479)	0.0000068 (0.00000368)	1.835	0.177
	alpha	0.0000140 (00001389)	0.0000136 (0.00001090)	0.094	0.759
	beta	0.0000047 (0.00000390)	0.0000030 (0.00000285)	16.501 ***	<0.001
	beta1	0.0000040 (0.00000304)	0.0000035 (0.00000228)	2.787	0.096
	beta2	0.0000055 (0.00000659)	0.0000040 (0.00000878)	2.471	0.117
	beta3	0.0000047 (0.00000439)	0.0000024 (0.00000231)	28.060 ***	<0.001
	high-beta	0.0000031 (0.00000287)	0.0000017 (0.00000168)	21.732 ***	<0.001
L-BA13p	delta	0.0000193 (0.00001338)	0.0000232 (0.00001275)	6.115 *	<0.05
	theta	0.0000082 (0.00000673)	0.0000090 (0.00000517)	1.259	0.263
	alpha	0.0000231 (0.00002368)	0.0000232 (0.00001901)	0.001	0.971
	beta	0.0000064 (0.00000541)	0.0000039 (0.00000252)	22.374 ***	<0.001
	beta1	0.0000064 (0.00000530)	0.0000054 (0.00000367)	2.948	0.085
	beta2	0.0000067 (0.00000638)	0.0000044 (0.00000400)	12.338 ***	<0.001
	beta3	0.0000062 (0.00000598)	0.0000031 (0.00000232)	32.102 ***	<0.001
	high-beta	0.0000039 (0.00000454)	0.0000019 (0.00000135)	23.756 ***	<0.001
R-BA13p	delta	0.0000131 (0.00000812)	0.0000176 (0.00000993)	16.580 ***	<0.001
	theta	0.0000062 (0.00000536)	0.0000068 (0.00000384)	1.140	0.287
	alpha	0.0000216 (0.00002237)	0.0000218 (0.00001829)	0.006	0.941
	beta	0.0000051 (0.00000428)	0.0000033 (0.00000225)	19.669 ***	<0.001
	beta1	0.0000049 (0.00000408)	0.0000044 (0.00000340)	1.244	0.266
	beta2	0.0000056 (0.00000560)	0.0000036 (0.00000350)	12.256 ***	0.001
	beta3	0.0000050 (0.00000459)	0.0000026 (0.00000208)	29.549 ***	<0.001
	high-beta	0.0000027 (0.00000241)	0.0000016 (0.00000133)	23.667 ***	<0.001
L-BA32	delta	0.0000159 (0.00001015)	0.0000227 (0.00001626)	16.972 ***	<0.001
	theta	0.0000046 (0.00000312)	0.0000055 (0.00000294)	6.737 **	0.010
	alpha	0.0000048 (0.00000366)	0.0000051 (0.00000322)	0.326	0.568
	beta	0.0000034 (0.00000329)	0.0000022 (0.00000273)	11.190 ***	0.001
	beta1	0.0000024 (0.00000171)	0.0000021 (0.00000129)	2.135	0.145
	beta2	0.0000036 (0.00000496)	0.0000033 (0.00000980)	0.130	0.718
	beta3	0.0000038 (0.00000432)	0.0000018 (0.00000208)	23.918 ***	<0.001
	high-beta	0.0000029 (0.00000307)	0.0000014 (0.00000174)	22.565 ***	<0.001
R-BA32	delta	0.0000155 (0.00000973)	0.0000225 (0.00001328)	25.067 ***	<0.001
	theta	0.0000045 (0.00000330)	0.0000054 (0.00000279)	5.575 *	<0.05
	alpha	0.0000048 (0.00000391)	0.0000049 (0.00000286)	0.024	0.876
	beta	0.0000036 (0.00000355)	0.0000024 (0.00000363)	7.277 **	<0.01
	beta1	0.0000024 (0.00000171)	0.0000022 (0.00000138)	1.901	0.169
	beta2	0.0000041 (0.00000747)	0.0000040 (0.00001373)	0.017	0.898
	beta3	0.0000038 (0.00000434)	0.0000018 (0.00000230)	22.283 ***	<0.001
	high-beta	0.0000028 (0.00000302)	0.0000015 (0.00000186)	20.001 ***	<0.001
**dlPFC**		**MDD group** **Mean (SD)**	**Healthy controls** **Mean (SD)**	***F***	***p***
L-BA8	delta	0.0000140 (0.00001081)	0.0000194 (0.00001890)	8.495 **	<0.01
	theta	0.0000047 (0.00000326)	0.0000056 (0.00000315)	5.838 *	<0.05
	alpha	0.0000054 (0.00000442)	0.0000058 (0.00000473)	0.540	0.463
	beta	0.0000039 (0.00000358)	0.0000025 (0.00000278)	12.572 ***	<0.001
	beta1	0.0000029 (0.00000213)	0.0000026 (0.00000173)	1.826	0.178
	beta2	0.0000037 (0.00000342)	0.0000032 (0.00000877)	0.274	0.601
	beta3	0.0000044 (0.00000478)	0.0000022 (0.00000251)	23.382 ***	<0.001
	high-beta	0.0000032 (0.00000329)	0.0000017 (0.00000181)	22.357 ***	<0.001
R-BA8	delta	0.0000126 (0.00000985)	0.0000177 (0.00001267)	13.711 ***	<0.001
	theta	0.0000046 (0.00000333)	0.0000055 (0.00000303)	5.284 *	<0.05
	alpha	0.0000055 (0.00000466)	0.0000057 (0.00000377)	0.149	0.700
	beta	0.0000040 (0.00000361)	0.0000026 (0.00000275)	11.803 **	0.001
	beta1	0.0000029 (0.00000217)	0.0000026 (0.00000172)	1.571	0.211
	beta2	0.0000039 (0.00000410)	0.0000036 (0.00000855)	0.183	0.669
	beta3	0.0000044 (0.00000474)	0.0000022 (0.00000254)	22.797 ***	<0.001
	high-beta	0.0000031 (0.00000321)	0.0000017 (0.00000185)	20.365 ***	<0.001
L-BA9	delta	0.0000168 (0.00001246)	0.0000234 (0.00002248)	9.166 **	<0.01
	theta	0.0000051 (0.00000352)	0.0000060 (0.00000329)	5.190 *	<0.05
	alpha	0.0000064 (0.00000527)	0.0000067 (0.00000531)	0.288	0.592
	beta	0.0000040 (0.00000360)	0.0000026 (0.00000268)	13.876 ***	<0.001
	beta1	0.0000030 (0.00000211)	0.0000027 (0.00000164)	2.053	0.153
	beta2	0.0000040 (0.00000378)	0.0000035 (0.00000855)	0.401	0.527
	beta3	0.0000045 (0.00000480)	0.0000022 (0.00000242)	24.858 ***	<0.001
	high-beta	0.0000033 (0.00000326)	0.0000017 (0.00000181)	25.316 ***	<0.001
R-BA9	delta	0.0000154 (0.00001138)	0.0000221 (0.00001326)	20.055 ***	<0.001
	theta	0.0000048 (0.00000370)	0.0000057 (0.00000301)	4.172 *	<0.05
	alpha	0.0000062 (0.00000539)	0.0000062 (0.00000411)	0.016	0.900
	beta	0.0000042 (0.00000378)	0.0000028 (0.00000367)	8.502 **	<0.01
	beta1	0.0000030 (0.00000204)	0.0000027 (0.00000169)	1.662	0.198
	beta2	0.0000046 (0.00000679)	0.0000044 (0.00001327)	0.039	0.844
	beta3	0.0000045 (0.00000477)	0.0000023 (0.00000264)	22.254 ***	<001
	high-beta	0.0000032 (0.00000326)	0.0000018 (0.00000195)	20.664 ***	<0.001
L-BA10	delta	0.0000210 (0.00001396)	0.0000314 (0.00002238)	21.191 ***	<0.001
	theta	0.0000057 (0.00000388)	0.0000067 (0.00000343)	5.020 *	< 0.05
	alpha	0.0000070 (0.00000678)	0.0000066 (0.00000479)	0.241	0.624
	beta	0.0000040 (0.00000393)	0.0000029 (0.00000514)	4.544	0.157
	beta1	0.0000029 (0.00000197)	0.0000026 (0.00000176)	1.399	0.238
	beta2	0.0000053 (0.00001128)	0.0000050 (0.00002009)	0.025	0.875
	beta3	0.0000040 (0.00000396)	0.0000021 (0.00000243)	23.594 ***	<0.001
	high-beta	0.0000032 (0.00000365)	0.0000017 (0.00000201)	17.85 ***	<0.001
R-BA10	delta	0.0000208 (0.00001390)	0.0000315 (0.00002064)	25.500 ***	<0.001
	theta	0.0000056 (0.00000426)	0.0000066 (0.00000339)	4.147 *	<0.05
	alpha	0.0000066 (0.00000637)	0.0000061 (0.00000409)	0.574	0.446
	beta	0.0000043 (0.00000447)	0.0000031 (0.00000597)	3.824	0.052
	beta1	0.0000030 (0.00000205)	0.0000027 (0.00000193)	1.701	0.193
	beta2	0.0000059 (0.00001361)	0.0000056 (0.00002325)	0.017	0.895
	beta3	0.0000043 (0.00000434)	0.0000022 (0.00000288)	20.924 ***	<0.001
	high-beta	0.0000033 (0.00000360)	0.0000018 (0.00000222)	16.964 ***	<0.001
L-BA46	delta	0.0000226 (0.00001671)	0.0000313 (0.00002522)	11.389 ***	0.001
	theta	0.0000062 (0.00000420)	0.0000073 (0.00000386)	4.999 *	<0.05
	alpha	0.0000088 (0.00000774)	0.0000091 (0.00000647)	0.072	0.789
	beta	0.0000047 (0.00000383)	0.0000031 (0.00000289)	15.811 ***	<0.001
	beta1	0.0000036 (0.00000244)	0.0000032 (0.00000198)	2.588	0.109
	beta2	0.0000051 (0.00000589)	0.0000043 (0.00000905)	−0.907	0.342
	beta3	0.0000050 (0.00000484)	0.0000025 (0.00000250)	27.705 ***	<0.001
	high-beta	0.0000038 (0.00000348)	0.0000019 (0.00000186)	28.878 ***	<0.001
R-BA46	delta	0.0000192 (0.00001370)	0.0000289 (0.00001786)	25.143 ***	<0.001
	theta	0.0000055 (0.00000424)	0.0000064 (0.00000352)	3.672	0.056
	alpha	0.0000085 (0.00000786)	0.0000084 (0.00000594)	0.012	0.914
	beta	0.0000047 (0.00000447)	0.0000033 (0.00000561)	5.155 *	<0.05
	beta1	0.0000033 (0.00000228)	0.0000030 (0.00000209)	1.397	0.238
	beta2	0.0000059 (0.00001197)	0.0000055 (0.00002152)	0.035	0.852
	beta3	0.0000048 (0.00000489)	0.0000025 (0.00000301)	21.364 ***	<0.001
	high-beta	0.0000036 (0.00000358)	0.0000020 (0.00000220)	18.397 ***	<0.001
**vlPFC**		**MDD group** **Mean (SD)**	**Healthy controls** **Mean (SD)**	***F***	***p***
L-BA44	delta	0.0000194 (0.00001494)	0.0000249 (0.00001920)	7.133 **	<0.01
	theta	0.0000065 (0.00000459)	0.0000075 (0.00000416)	3.707	0.055
	alpha	0.0000119 (0.00001122)	0.0000124 (0.00001008)	0.171	0.679
	beta	0.0000054 (0.00000450)	0.0000034 (0.00000251)	20.543 ***	<0.001
	beta1	0.0000047 (0.00000364)	0.0000041 (0.00000262)	2.789	0.096
	beta2	0.0000055 (0.00000472)	0.0000041 (0.00000514)	5.266 *	<0.05
	beta3	0.0000057 (0.00000549)	0.0000028 (0.00000262)	29.964 ***	<0.001
	high-beta	0.0000040 (0.00000369)	0.0000020 (0.00000165)	32.338 ***	<0.001
R-BA44	delta	0.0000146 (0.00001076)	0.0000206 (0.00001203)	18.749 ***	<0.001
	theta	0.0000052 (0.00000407)	0.0000060 (0.00000330)	3.523	0.062
	alpha	0.0000107 (0.00001102)	0.0000110 (0.00000830)	0.048	0.827
	beta	0.0000047 (0.00000397)	0.0000031 (0.00000284)	13.636 ***	<0.001
	beta1	0.0000039 (0.00000293)	0.0000035 (0.00000237)	1.217	0.271
	beta2	0.0000050 (0.00000562)	0.0000041 (0.00000833)	1.143	0.286
	beta3	0.0000049 (0.00000482)	0.0000026 (0.00000252)	24.739 ***	<0.001
	high-beta	0.0000032 (0.00000301)	0.0000018 (0.00000174)	22.156 ***	<0.001
L-BA45	delta	0.0000247 (0.00001854)	0.0000329 (0.00002351)	10.431 ***	0.001
	theta	0.0000072 (0.00000502)	0.0000083 (0.00000449)	3.721	0.055
	alpha	0.0000118 (0.00001117)	0.0000118 (0.00000883)	0.002	0.962
	beta	0.0000054 (0.00000416)	0.0000034 (0.00000256)	21.942 ***	<0.001
	beta1	0.0000045 (0.00000319)	0.0000039 (0.00000243)	3.671	0.056
	beta2	0.0000058 (0.00000546)	0.0000043 (0.00000625)	4.324 *	<0.05
	beta3	0.0000056 (0.00000506)	0.0000028 (0.00000255)	31.390 ***	<0.001
	high-beta	0.0000042 (0.00000372)	0.0000021 (0.00000184)	31.923 ***	<0.001
R-BA45	delta	0.0000196 (0.00001453)	0.0000286 (0.00001763)	21.261 ***	<0.001
	theta	0.0000059 (0.00000456)	0.0000068 (0.00000387)	3.210	0.074
	alpha	0.0000116 (0.00001099)	0.0000116 (0.00000882)	0.001	0.977
	beta	0.0000051 (0.00000437)	0.0000035 (0.00000432)	9.638 **	<0.01
	beta1	0.0000040 (0.00000281)	0.0000035 (0.00000243)	1.684	0.195
	beta2	0.0000061 (0.00000952)	0.0000051 (0.00001511)	0.455	0.501
	beta3	0.0000052 (0.00000501)	0.0000028 (0.00000299)	23.181 ***	<0.001
	high-beta	0.0000037 (0.00000352)	0.0000021 (0.00000212)	19.710 ***	<0.001
L-BA47	delta	0.0000246 (0.00001581)	0.0000324 (0.00001873)	13.959 ***	<0.001
	theta	0.0000081 (0.00000613)	0.0000089 (0.00000473)	1.405	0.237
	alpha	0.0000160 (0.00001836)	0.0000144 (0.00001261)	0.698	0.404
	beta	0.0000054 (0.00000411)	0.0000034 (0.00000265)	23.803 ***	<0.001
	beta1	0.0000050 (0.00000349)	0.0000041 (0.00000248)	5.038 *	<0.05
	beta2	0.0000063 (0.00000653)	0.0000044 (0.00000819)	4.399 *	<0.05
	beta3	0.0000053 (0.00000460)	0.0000026 (0.00000207)	37.230 ***	<0.001
	high-beta	0.0000038 (0.00000346)	0.0000019 (0.00000156)	31.859 ***	<0.001
R-BA47	delta	0.0000187 (0.00001175)	0.0000271 (0.00001581)	25.040 ***	<0.001
	theta	0.0000063 (0.00000495)	0.0000071 (0.00000390)	1.981	0.160
	alpha	0.0000124 (0.00001247)	0.0000117 (0.00000968)	0.334	0.564
	beta	0.0000049 (0.00000417)	0.0000033 (0.00000428)	10.160 **	<0.01
	beta1	0.0000040 (0.00000288)	0.0000034 (0.00000224)	3.028	0.083
	beta2	0.0000061 (0.00000950)	0.0000049 (0.00001567)	0.561	0.455
	beta3	0.0000048 (0.00000451)	0.0000025 (0.00000261)	26.151 ***	<0.001
	high-beta	0.0000033 (0.00000325)	0.0000019 (0.00000190)	20.178 ***	<0.001
**OFC**		**MDD group** **Mean (SD)**	**Healthy controls** **Mean (SD)**	***F***	***p***
L-BA10	delta	0.0000210 (0.00001396)	0.0000314 (0.00002238)	21.191 ***	<0.001
	theta	0.0000057 (0.00000388)	0.0000067 (0.00000343)	5.020 *	<0.05
	alpha	0.0000070 (0.00000678)	0.0000066 (0.00000479)	0.241	0.624
	beta	0.0000040 (0.00000393)	0.0000029 (0.00000514)	4.544	0.157
	beta1	0.0000029 (0.00000197)	0.0000026 (0.00000176)	1.399	0.238
	beta2	0.0000053 (0.00001128)	0.0000050 (0.00002009)	0.025	0.875
	beta3	0.0000040 (0.00000396)	0.0000021 (0.00000243)	23.594 ***	<0.001
	high-beta	0.0000032 (0.00000365)	0.0000017 (0.00000201)	17.85 ***	<0.001
R-BA10	delta	0.0000208 (0.00001390)	0.0000315 (0.00002064)	25.500 ***	<0.001
	theta	0.0000056 (0.00000426)	0.0000066 (0.00000339)	4.147 *	<0.05
	alpha	0.0000066 (0.00000637)	0.0000061 (0.00000409)	0.574	0.446
	beta	0.0000043 (0.00000447)	0.0000031 (0.00000597)	3.824	0.052
	beta1	0.0000030 (0.00000205)	0.0000027 (0.00000193)	1.701	0.193
	beta2	0.0000059 (0.00001361)	0.0000056 (0.00002325)	0.017	0.895
	beta3	0.0000043 (0.00000434)	0.0000022 (0.00000288)	20.924 ***	<0.001
	high-beta	0.0000033 (0.00000360)	0.0000018 (0.00000222)	16.964 ***	<0.001
L-BA11	delta	0.0000220 (0.00001220)	0.0000297 (0.00001663)	19.136 ***	<0.001
	theta	0.0000074 (0.00000577)	0.0000080 (0.00000434)	0.969	0.326
	alpha	0.0000145 (0.00001754)	0.0000127 (0.00001219)	1.006	0.317
	beta	0.0000048 (0.00000385)	0.0000030 (0.00000346)	16.495 ***	<0.001
	beta1	0.0000043 (0.00000305)	0.0000036 (0.00000215)	5.429 *	<0.05
	beta2	0.0000060 (0.00000806)	0.0000043 (0.00001287)	1.622	0.204
	beta3	0.0000046 (0.00000409)	0.0000022 (0.00000190)	35.659 ***	<0.001
	high-beta	0.0000032 (0.00000333)	0.0000016 (0.00000148)	25.599 ***	<0.001
R-BA11	delta	0.0000196 (0.00001077)	0.0000277 (0.00001535)	25.469 ***	<0.001
	theta	0.0000069 (0.00000550)	0.0000075 (0.00000408)	1.195	0.275
	alpha	0.0000128 (0.00001469)	0.0000112 (0.00001047)	1.000	0.318
	beta	0.0000047 (0.00000398)	0.0000030 (0.00000373)	12.939 ***	<0.001
	beta1	0.0000040 (0.00000288)	0.0000033 (0.00000207)	4.656	0.031
	beta2	0.0000059 (0.00000913)	0.0000045 (0.00001368)	1.053	0.306
	beta3	0.0000044 (0.00000419)	0.0000022 (0.00000225)	29.511 ***	<0.001
	high-beta	0.0000031 (0.00000334)	0.0000016 (0.00000165)	20.062 ***	<0.001
**PCC**		**MDD group** **Mean (SD)**	**Healthy controls** **Mean (SD)**	***F***	***p***
L-BA23	delta	0.0000076 (0.00000468)	0.0000092 (0.00000503)	7.375 **	<0.01
	theta	0.0000054 (0.00000476)	0.0000060 (0.00000371)	1.231	0.268
	alpha	0.0000261 (0.00002698)	0.0000272 (0.00002387)	0.127	0.722
	beta	0.0000050 (0.00000452)	0.0000033 (0.00000249)	14.819 ***	<0.001
	beta1	0.0000058 (0.00000540)	0.0000055 (0.00000506)	0.196	0.659
	beta2	0.0000050 (0.00000614)	0.0000031 (0.00000300)	10.111 **	<0.01
	beta3	0.0000047 (0.00000448)	0.0000024 (0.00000199)	28.442 ***	< 0.001
	high-beta	0.0000021 (0.00000191)	0.0000011 (0.00000091)	29.532 ***	<0.001
R-BA23	delta	0.0000075 (0.00000456)	0.0000093 (0.00000502)	7.375 **	<0.01
	theta	0.0000054 (0.00000476)	0.0000060 (0.00000366)	1.231	0.268
	alpha	0.0000263 (0.00002715)	0.0000272 (0.00002382)	0.127	0.771
	beta	0.0000050 (0.00000442)	0.0000033 (0.00000244)	14.819 ***	<0.001
	beta1	0.0000057 (0.00000521)	0.0000054 (0.00000501)	0.196	0.669
	beta2	0.0000049 (0.00000598)	0.0000031 (0.00000294)	10.111 ***	0.001
	beta3	0.0000046 (0.00000441)	0.0000024 (0.00000194)	28.442 ***	<0.001
	high-beta	0.0000021 (0.00000188)	0.0000011 (0.00000090)	29.532 ***	<0.001
L-BA31	delta	0.0000079 (0.00000598)	0.0000093 (0.00000587)	3.887 *	0.050
	theta	0.0000051 (0.00000437)	0.0000056 (0.00000353)	1.053	0.306
	alpha	0.0000254 (0.00002595)	0.0000264 (0.00002358)	0.099	0.753
	beta	0.0000049 (0.00000449)	0.0000033 (0.00000253)	13.153 ***	<0.001
	beta1	0.0000056 (0.00000535)	0.0000054 (0.00000499)	0.106	0.745
	beta2	0.0000050 (0.00000622)	0.0000032 (0.00000303)	8.904 **	<0.01
	beta3	0.0000046 (0.00000446)	0.0000025 (0.00000202)	25.719 ***	<0.001
	high-beta	0.0000021 (0.00000181)	0.0000011 (0.00000091)	27.424 ***	<0.001
R-BA31	delta	0.0000078 (0.00000569)	0.0000094 (0.00000586)	5.143 *	<0.05
	theta	0.0000051 (0.00000438)	0.0000056 (0.00000350)	1.119	0.291
	alpha	0.0000257 (0.00002617)	0.0000264 (0.00002343)	0.055	0.814
	beta	0.0000049 (0.00000439)	0.0000033 (0.00000248)	13.504 ***	<0.001
	beta1	0.0000056 (0.00000517)	0.0000054 (0.00000495)	0.094	0.760
	beta2	0.0000049 (0.00000606)	0.0000031 (0.00000297)	9.306 **	<0.01
	beta3	0.0000046 (0.00000438)	0.0000024 (0.0000042)	26.250 ***	<0.001
	high-beta	0.0000020 (0.00000178)	0.0000011 (0.00000088)	26.837 ***	<0.001

* *p* < 0.05, ** *p* < 0.01, *** *p* < 0.001 L, left; R, right; dmPFC, dorsal medial prefrontal cortex (including BA8, BA9); vmPFC, ventral medial prefrontal cortex (including BA10, BA11, BA13, BA32); dlPFC, dorsal lateral prefrontal cortex (including BA8, BA9, BA10, BA46); vlPFC, ventral lateral prefrontal cortex (including BA44, BA45, BA47); OFC, orbital frontal cortex (including BA10, BA11); PCC, posterior cingulate cortex (including BA23, BA31).

## Data Availability

The data from this study are available from the corresponding author upon reasonable request.
